# The Orphan Receptor GPR151: Discovery, Expression, and Emerging Biological Significance

**DOI:** 10.1021/acschemneuro.4c00780

**Published:** 2025-04-28

**Authors:** Olivia DePasquale, Chris O’Brien, Baila Gordon, David J. Barker

**Affiliations:** † Department of Psychology, 242612 Rutgers, The State University of New Jersey, 152 Frelinghuysen Road, Piscataway, New Jersey 08854, United States; ‡ Brain Health Institute, Rutgers University, Piscataway, New Jersey 08854, United States; § Rutgers Addiction Research Center, Piscataway, New Jersey 08854, United States

**Keywords:** Pain, Addiction, Ingestion, Orphan, GPCR, habenula

## Abstract

G protein-coupled receptors (GPCRs) are among the most prominent druggable targets in the human genome, accounting for approximately 40% of marketed drugs. Despite this, current GPCR-targeted therapies address only about 10% of the GPCRs encoded in the genome. Expanding our knowledge of the remaining “orphan” GPCRs represents a critical frontier in drug discovery. GPR151 emerges as a compelling target due to its distinct expression in the habenula complex, spinal cord neurons, and dorsal root ganglia. This receptor is highly conserved across mammals and possesses orthologs in species such as zebrafish and chickens, underscoring its evolutionarily conserved role in fundamental mammalian processes. Although the precise function of GPR151 remains unknown, it has been strongly implicated in pain modulation and reward-seeking behavior. These attributes position GPR151 as a promising candidate for the development of targeted and specialized pharmacological therapies. This review summarizes the current literature on GPR151, including its discovery, structure, mechanisms, anatomical distribution, and functional roles, while also exploring potential directions for future research.

## Graphical abstract

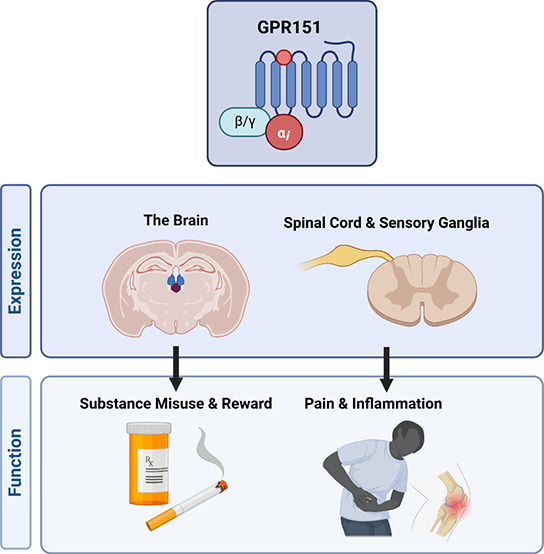

## Introduction

G protein-coupled receptors (GPCRs) constitute the largest family of receptor proteins encoded in the human genome, with approximately 800 members, accounting for about 3% of all genes.^1^ These ubiquitous cell-surface receptors detect environmental stimuli, such as light and odors, and play a crucial role in intercellular communication via hormones and neurotransmitters. GPCR activation triggers intracellular signaling cascades that regulate cellular metabolism, secretion, growth, and immune responses. GPCRs represent about 40% of all druggable targets in the human genome, making them one of the most widely targeted receptor families in pharmacology.^2–4^ Notable examples of GPCR-targeted therapeutics include semaglutide (a GLP1R agonist for obesity and diabetes), olanzapine (a D2 and 5HT-2A antagonist for schizophrenia and depression), and morphine (a mu-opioid receptor antagonist for chronic pain and analgesia).^2^ Several factors contribute to the widespread utility of GPCR-targeted drugs, including their accessibility as cell-surface receptors, interactions with multiple signaling pathways, and regulation of diverse physiological processes.^2^ However, despite their therapeutic potential, current GPCR-targeted drugs only focus on about 80 known receptors—roughly 10% of all GPCRs encoded in the human genome.^5^ Elucidating the structure, biology, and therapeutic effects of the remaining GPCRs, particularly the “orphan” receptors with unidentified endogenous ligands, remains a compelling force in GPCR-based drug discovery.

Among orphan GPCRs, GPR151 (also known as GPCR-2037, GALRL, GALR4, and PGR7) stands out as a receptor of particular interest. While GPCRs often exhibit widespread expression patterns in the brain, GPR151 expression is primarily restricted to the habenula complex (Hb), spinal cord neurons, and dorsal root ganglia (DRG).^6–8^ The habenula complex plays a key role in substance misuse, reinforcement learning, decision-making, and pain processing.^9–12^ Meanwhile, the spinal cord and DRG are essential for communication between the peripheral and central nervous systems, coordinating motor commands, reflexes, and sensory information processing.^13,14^ Its distinct and localized expression suggests specialized functions within these neural circuits, reinforcing its potential as a key target for further study.

The GPR151 gene is highly conserved across mammals (∼83% identity between humans and rodents; ∼89% between rats and mice) and has orthologs in zebrafish (54% DNA sequence identity with humans) and chickens.^9,15^ Although GPR151 is mapped to different chromosomes across species, it contains a conserved CpG dinucleotide region with 10 CpG sites near the transcriptional start site.^16^ This preserved genomic sequence across vertebrates suggests a fundamental role in mammalian function.

Overall, GPR151 represents a promising target for the development of localized and specialized pharmacological interventions. This review aims to summarize the current literature on GPR151, covering its discovery, structure, mechanisms, and anatomical expression. Additionally, we highlight its known roles in pain processing and reward-seeking behavior and explore potential implications in obesity and cancer. Finally, we discuss future research directions and therapeutic opportunities.

## Structure, Ligand, and Signaling Pathway of GPR151

GPR151 was first identified due to its homology with galanin receptors.^6,8^ At the amino acid level, GPR151 shares 25–26% identity and 41–43% similarity with the galanin receptor family.^6,8,17,18^ Sequence-structure phylogenetic analysis classified GPR151 within “cluster 14” of the rhodopsin-like family, grouping it alongside galanin receptors (GalR1, GalR2, GalR3) and the kisspeptin receptor (Kiss1R/Gpr54).^18^ Broader sequence analysis categorized GPR151 within the “SOG” subfamily, which includes somatostatin, opioid, galanin, and kisspeptin receptors.^18–22^ The SOG receptor family is a subset of Class A GPCRs—the largest, most diverse, and best-characterized GPCR cluster in humans. Class A receptors share a characteristic seven-transmembrane (7TM) helix domain, featuring a ligand-binding pocket and a G-protein-binding region located at the extracellular and intracellular ends of the helix bundle. However, some studies suggest that GPR151’s classification within the rhodopsin-like family remains ambiguous due to variations in findings.^20–22^ While the structure of GPR151 has been inferred from its similarity to other SOG receptors and modeling approaches,^23^ it has not yet been experimentally validated. As of this review, the crystal structure of GPR151 has yet to be solved.

Despite the unresolved structure, efforts to deorphan GPR151 have been undertaken. Due to its strong homology with the galanin receptor family, researchers initially hypothesized that GPR151 might respond to the neuropeptides galanin and kisspeptin. However, GPR151 exhibits weak activation by galanin (EC50 of 2 μM) or shows no activation at all.^8,24,25^ Furthermore, its potential interaction with kisspeptins remains unexplored. Thus, although GPR151’s endogenous ligand may share structural features with galanin, current evidence suggests that galanin itself is unlikely to be the native ligand. Given this, efforts to identify GPR151’s ligand are ongoing.

Mashiko and colleagues proposed that protons might act as ligands, as GPR151 showed significantly enhanced activation and gene expression in cultured cells following changes in extracellular pH (from 6 to 5).^26^ Notably, proton-sensitive GPCRs are rare, with only three well-characterized members—GPR4, GPR65, and GPR68.^27^ Interestingly, GPR68 lacks a specific proton-binding domain but instead relies on a network of residues for activation. Determining GPR151’s crystal structure could reveal if it shares this mechanism and clarify the influence of acidic environments on its activation in vivo. In the absence of a crystal structure, deep learning models have been used to predict receptor structures and identify potential ligands for orphan receptors.^28^ However, these predictions have yet to be validated in vivo, and no definitive ligand for GPR151 has been identified to date.

Due to the lack of a resolved structure and identified ligand, the precise mechanism of GPR151 remains largely unknown. In the brain, GPR151 localizes to presynaptic membranes and synaptic vesicles, associating with presynaptic proteins involved in neurotransmitter release.^29^ These findings suggest that GPR151 functions presynaptically, modulating synaptic vesicle release under basal conditions.^29^ Upon activation, GPR151 is believed to couple with the heterotrimeric Gαo1 subunit and interact with synaptic components that control ion transport, such as the sodium/potassium pump.^29^ This coupling results in the dissociation of the active Gβγ subunit, subsequently activating the extracellular signal-regulated kinase (ERK) pathway.^30^ GPR151 is thought to act presynaptically to influence the release probability of synaptic vesicles through coupling cAMP signaling to neurotransmitter release machinery;^29,30^ however, as only a limited number of studies have contributed to this research,^29,30^ further investigations are required to establish a thorough understanding of GR151’s mechanism. Based on its regulation of cAMP measured with a lance cAMP assay, findings from Antolin-Fontes and colleagues (2020) suggest that GPR151 may be constitutively active.^29^ However, Kroeze et al. assessed the constitutive activity of nearly all nonolfactory GPCRs using a Tango β-arrestin assay and reported that GPR151 exhibited one of the lowest constitutive activity levels among GPCRs studied.^31^ The Tango β-arrestin assay is arrestin dependent, while the Lance cAMP assay involves Gs-coupling. Thus, the data from these studies may not conflict, but rather suggest nuanced signaling properties of GPR151, possibly due to its unique structure, allowing for biased signaling and/or coupling to G-proteins rather than β-arrestin. It will be important to reconcile these studies to fully elucidate GPR151’s mechanism and structure, as they carry important implications for identifying putative ligands for GPR151, and for fully elucidating its function.

## Expression of GPR151

### Expression of GPR151 in the Developing and Mature Brain

In mouse embryonic development, GPR151 mRNA is primarily expressed in the nervous system. By embryonic day 10.5, GPR151 mRNA expression emerges in a small group of cells at the hindbrain–brain boundary.^8^ By embryonic day 14.5, GPR151 mRNA is highly expressed in the dorsal median thalamus and locus coeruleus, with lower levels observed in motoric hindbrain nuclei, including the facial and hypoglossal nerve nuclei.^8^ At later stages, GPR151 mRNA is detected in the anterior amygdala, caudate putamen, and superficial layers of the superior and inferior colliculi.^8^ However, the paraventricular nucleus and the dorsal median thalamus, particularly the habenular region, exhibit the highest GPR151 mRNA expression.^8^ This pattern continues in the mature brain where the highest accumulation of transcripts is in the habenula complex.^32^

GPR151 mRNA exhibits remarkable specificity for the habenular complex. GPR151 is the most selectively expressed gene in the habenula, with minimal expression in other regions of both adult and fetal human brains.^6,8,32–36^ The habenula is a structurally heterogeneous region, comprising up to 15 subnuclei.^37–40^ In the medial habenula, GPR151 is robustly expressed in the lateral division and cholinergic ventral medial habenula (Figure 1A).^6,8,32^ In the lateral habenula, *GPR151* mRNA and protein expression has been observed in all subnuclei, except the oval subnucleus, but the parvocellular and central subnuclei of the lateral division stand out for prominent GPR151 protein expression (Figure 1A).^9^

**Figure 1:**
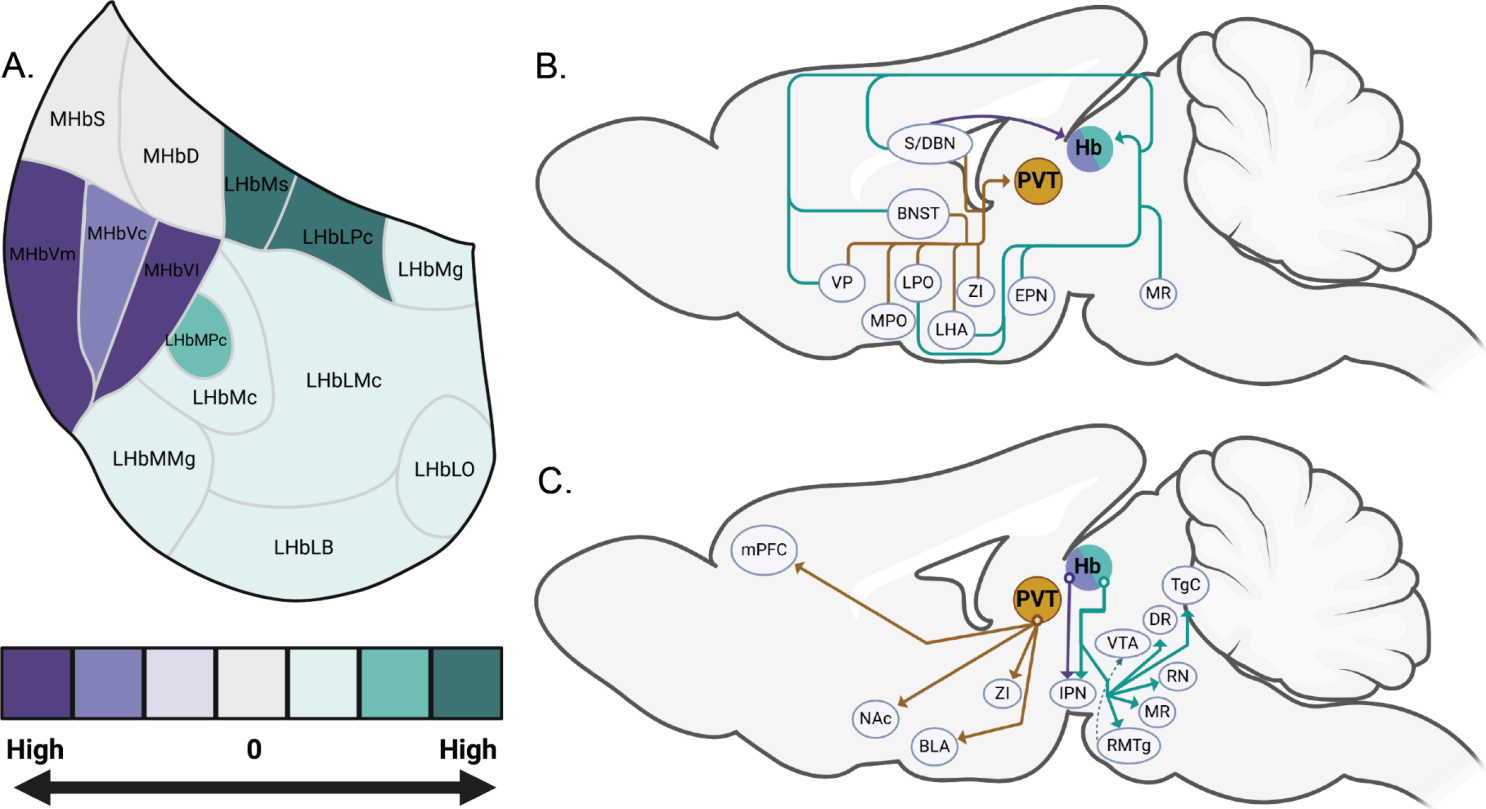
GPR151 expression pattern in habenular subnuclei and the afferent and efferent circuitry of GPR151-expressing neurons. (A) Schematic coronal section detailing the expression pattern of GPR151 receptor expression within habenular subnuclei. General level of expression is demonstrated in saturation of color. Within the medial habenula, shown in purple, GPR151 expression is primarily limited to the MHbVm and MHbVI. In the lateral habenula, shown in green, expression is greatest in the LHbMs and LHbLPc, with some expression in the LHbMPc and across the entire lateral habenula. (B) Schematic sagittal brain section demonstrating outputs of the paraventricular thalamus in orange, the medial habenula in purple, and the lateral habenula in green. (C) Schematic sagittal brain section demonstrating inputs to the paraventricular thalamus in orange, the medial habenula in purple, and the lateral habenula in green. It should be noted that thalamic GPR151 in B,C corresponds to cells expressing GPR151 mRNA but not protein. Abbreviations: BLA, basolateral amygdala; BNST, bed nucleus of the stria terminalis; DR, dorsal raphe nucleus; EPN, entopeduncular nucleus; IPN; interpeduncular nucleus; LHA, lateral hypothalamic area; LHb, lateral habenula; LHbLMc, magnocellular part of the lateral LHb; LHbLO, oval part of the LHbL; LHbLPc, parvocellular subnucleus of the LHbL; LHbMC, central part of the medial LHb; LHbMMg, marginal part of the LHbM; LHbMPc, parvocellular subnucleus of the LHbM; LHbMS, superior part of the LHbM; LPO, lateral preoptic area; mHb, medial habenula; MHbCd, central and dorsal part of the MHb; MHbCv, central and ventral part of the MHb; MHbI, inferior part of the MHb; MHbS, superior part of the MHb; MHbVL, ventral and lateral part of the MHb; mPFC, medial prefrontal cortex; MR, medial raphe nucleus; NAc, nucleus accumbens; PVT, paraventricular thalamic nucleus; RN, rhabdoid nucleus; RMTg, rostromedial tegmental nucleus; S/DBN, septum and diagonal band nuclei; TgC, Tegmentum; VP, ventral pallidum; VTA, ventral tegmental area; ZI, zona incerta.

The habenula’s heterogeneity is reflected in the diverse connectivity of GPR151-positive neurons. Inputs to GPR151-Cre positive neurons from the posterior septum and diagonal band likely target the medial habenula, while inputs from the lateral preoptic area, lateral hypothalamus, pallidum, and stria terminalis likely target the LHb, as these are traditional inputs to the MHb and LHb (Figure 1B).^41,42^ The specific expression of GPR151 has also been seen in efferent fibers to many traditional habenular outputs. GPR151-positive fibers have been identified in the raphe nucleus, RMTg, dorsal tegmental nucleus, and lateral interpeduncular nucleus (Figure 1C). These fibers likely originate from the lateral habenula,^9,41,43,44^ while projections to the central interpeduncular nucleus are thought to arise from the medial habenula (Figure 1C).^41,45,46^ Interestingly, GPR151-positive fibers do not extend to the ventral tegmental area—a major output of the lateral habenula (Figure 1C).^9^ This suggests a GPR151-specific connectivity pattern. Notably, protein expression of GPR151 is limited to habenular neurons targeting the interpeduncular nucleus, the rostromedial tegmental nucleus, the rhabdoid nucleus, the median raphe, the caudal dorsal raphe, and the dorsal tegmentum in rats and mice.^9^

Beyond the habenula, low levels of GPR151 mRNA are detected in thalamic regions, including the paraventricular, reuniens, rhomboid, central lateral, and perifascicular nuclei.^8,39^ GPR151-Cre positive neurons in the paraventricular nucleus receive afferents from the pallidum, lateral and medial preoptic area, zona incerta, and peduncular part of the lateral hypothalamus (Figure 1B).^42^ It is thought that these GPR151-Cre positive paraventricular nucleus neurons project to the prelimbic area, the shell and core regions of the nucleus accumbens, the basolateral amygdala, and the zona incerta (Figure 1C).^42^ Furthermore, GPR151-Cre positive neurons of the lateral posterior thalamic nucleus receive afferents from the cingulate cortical areas 1 and 2, retrosplenial dysgranular cortex, primary and secondary visual cortex, and primary and secondary somatosensory cortex, which could indicate a role for GPR151 in processing visual information, as well as a related sensory or associative process.^42^ Strikingly, these regions exhibit little to no GPR151 protein immunoreactivity,^9^ suggesting that the observed mRNA expression may reflect ectopic developmental transcription. However, we cannot yet exclude the possibility that these neurons may express the GPR151 protein under specific circumstances.

The restricted expression of GPR151 protein within the habenula complex suggests region-specific functions and highlights its promise as a druggable target. Although the medial and lateral habenula are traditionally viewed as functionally distinct, the presence of GPR151 in both subdivisions suggests a potential unified functional system.

### Expression of GPR151—Spinal Cord and Periphery

Beyond its expression in the central nervous system, GPR151 mRNA is also detected in sensory ganglia of the peripheral nervous system during mouse embryonic development.^8,30^ GPR151 receptor proteins are restricted to a subpopulation of small-diameter, nonpeptidergic C-fiber neurons in the dorsal root ganglia (DRG)^47^ and trigeminal ganglia,^30^ while GPR151 mRNA is also detected in the vestibular ganglion.^8^ Additionally, GPR151 mRNA is predominantly expressed in excitatory and inhibitory neurons of laminae I–IV in the dorsal horn.^7,8,47^ In the DRG, trigeminal ganglia, and spinal cord, GPR151-positive cells are primarily neurons, with minimal expression in glial cells.^16,30^ GPR151 transcript expression remains stable throughout development, showing no significant changes in levels.^8^

Low levels of GPR151 mRNA have also been detected in peripheral tissues, including the kidney, liver, and pancreas.^8,48^ Ignatov hypothesized that the peripheral detection of GPR151 mRNA in Northern blot analyses may result from increased mRNA turnover.^8^ This suggests that while GPR151 is transcribed, it may not be translated into protein or may only be translated at minimal levels compared to the CNS. It remains unclear whether the detected mRNA originates from liver cells or from afferent projections of CNS neurons. To date, no studies have demonstrated functional GPR151 protein expression in the liver or other peripheral organs, raising questions about its physiological relevance. Further studies are needed to confirm and characterize the source of this peripheral expression.

## Potential Roles of GPR151

As an orphan GPCR, GPR151 has been investigated for its potential roles in pain processing, reward, cognition, obesity, and cancer, as discussed below.

### Pain Processing

GPR151 is selectively expressed in the habenula, sensory ganglion, and spinal cord, regions crucial for nociception and pain processing. This has led researchers to investigate GPR151’s potential role in pain modulation. Multiple studies report a marked increase in GPR151 mRNA expression in the dorsal root ganglion (DRG) and spinal cord following induction of neuropathic,^7,16,24,49,50^ inflammatory,^51^ or burn injury-induced pain.^52^ Although the trigeminal ganglion shows low basal expression of GPR151 mRNA, its expression is upregulated under neuropathic pain conditions.^30^ Additionally, *GPR151* mRNA in the trigeminal ganglia moderately colocalizes with calcitonin gene-related peptide (CGRP), a neuropeptide implicated in pain transmission.^9^ Given its consistent upregulation in pain states, several studies have examined the role of GPR151 in neuropathic pain. Reduction or deletion of *GPR151* attenuates neuropathic pain, while upregulation of *GPR151* induces neuropathic pain, without affecting basal nociception.^7,30,47^ These findings suggest that GPR151 is a key regulator of pain processing, prompting further research into its physiological function in pain modulation and recovery.

#### Molecular Mechanisms of GPR151 in Neuropathic Pain

Under neuropathic pain conditions, *GPR151* expression is regulated by DNA methyltransferase 3b (DNMT3b)-mediated DNA demethylation and Krüppel-like factor 5 (KLF5)-mediated transcriptional activation.^16^ DNA methyltransferases (DNMTs) are important in regulating DNA methylation and directly inhibit transcription by interfering with transcription factor binding.^53^ Consequently, inhibition of DNMT3b increases *GPR151* mRNA expression, reduces methylation of the GPR151 promoter, and induces mechanical allodynia.^16^ Krüppel-like factors (KLFs) are zinc-finger DNA-binding transcription factors that control a wide variety of biological processes.^54^ KLF5 facilitates the upregulation of GPR151 and demonstrates increased occupancy in the *GPR151* promoter in mice with neuropathic pain.^16^ In contrast, deletion of KLF5 reduces *GPR151* expression and attenuates neuropathic pain.^16^

GPR151 may also participate in neuropathic pain through activation of the mitogen-activated protein kinase/extracellular signal-regulated kinases (MAPK/ERK) signaling pathway.^16^ This pathway plays a vital role in the pathogenesis of chronic pain^55,56^ by initiating an increase in pain-related gene expression.^16^ Deletion of *GPR151* reduces ERK-dependent expression of several neuroinflammation-related genes^16,30^ and decreases neuronal excitability in trigeminal ganglia neurons following induction of neuropathic pain.^30^ This suggests that GPR151 drives neuropathic pain by activating Gβγ/ERK signaling pathways, which in turn upregulate algogenic gene expression, increase neuronal excitability, and amplify pain responses.^16,30^

GPR151 also contributes to neuropathic pain through its regulation of microglial activation. In males, microglial activation is both necessary and sufficient for central sensitization and neuropathic pain.^57–61^ Colony-stimulating factor 1 (CSF1), a primary growth factor necessary for microglial proliferation in the spinal cord following nerve injury, is upregulated in GPR151-positive DRG neurons upon neuropathic injury.^47,62^ This suggests that GPR151 promotes microglial proliferation, contributing to pain hypersensitivity. One possible mechanism involves P2X3 receptors, ATP-gated ion channels that mediate pain signaling and central sensitization.^17,63–66^ After nerve injury, both GPR151 and P2X3 are upregulated in nociceptive C-fiber DRG neurons.^47^ Overexpression of *GPR151* significantly enhanced P2X3-mediated calcium elevation and dorsal root ganglion excitability.^47^ Conversely, DRG-specific deletion of *GPR151* inhibits P2X3 function, reducing calcium elevation, DRG neuronal hyperexcitability, and CSF1 expression. This leads to normalized microglial activation and attenuation of chronic constriction injury-induced neuropathic pain.^47^ However, since these findings are based on studies conducted exclusively in male mice, further research is needed to determine whether GPR151’s role in pain processing differs across sexes.

Overall, there is compelling evidence that GPR151 plays an important role in pain circuitry through multiple distinct molecular mechanisms. Of note, Holmes et al. found that reducing GPR151 did not alleviate neuropathic pain-related behavior.^24^ This discrepancy may arise from differences in gene mutation strategies or pain induction methodologies. Nevertheless, most recent studies suggest that GPR151 plays a role in neuropathic pain, primarily through MAPK/ERK activation, transcriptional regulation, and microglial-mediated inflammation. Future research should focus on validating these findings in female cohorts and exploring potential therapeutic interventions targeting GPR151 in pain disorders.

### Reward-Seeking and Motivation

The expression pattern of GPR151 in the habenula and reward-related circuits suggests a role in motivated behavior. GPR151-positive fibers originating in the habenular complex innervate the dorsal raphe, a region implicated in affective and motivational processing,^9^ as well as other reward-related areas, including the zona incerta, medial preoptic area, and nucleus accumbens.^42^ Within the habenula, GPR151 also exhibits high colocalization with the mu-opioid receptor (OPRM1), which is critical for morphine’s analgesic effects.^35^

Although studies on GPR151’s role in motivation are limited, recent evidence links it to nicotine addiction. The medial habenula, a key regulator of nicotine intake, aversion, withdrawal, and relapse, densely expresses nicotinic acetylcholine receptors.^67^ GPR151 knockout mice exhibit increased self-administration of high nicotine doses, reduced aversion to high doses of nicotine, and diminished behavioral adaptation to repeated nicotine exposure, without baseline changes in affective behavior.^29^ These findings suggest that GPR151, likely acting through the medial habenula, negatively regulates nicotine reward and maintains homeostatic control over motivational and aversive states.

GPR151 may also influence reward salience. Mice with ablated GPR151-positive neurons show normal reward valuation at baseline but exhibit strong reward devaluation when tasks require delays or increased effort.^46^ Additionally, GPR151 deletion reduced social behaviors in social preference tests, though it remains unclear whether this stems from more general changes in negative affective behaviors or cognitive function.^68^ Supporting this, deletion of GPR151 Cre-positive neurons has been linked to hyperactivity, impaired spatial memory, deficits in flexible learning, and increased impulsive and compulsive behaviors.^46^

While emerging evidence highlights GPR151’s involvement in reward, motivation, and nicotine addiction, much remains to be explored. Its selective expression in the habenula, connections to key reward-related regions, and interactions with neuromodulatory systems position it as a potential regulator of reward salience and aversive learning. Current findings suggest that GPR151 may act as a negative regulator of reward pathways and motivation. However, its broader influence on cognition, impulsivity, and social behavior underscores the need for future research. Given the habenula’s established role in negative reward prediction error, future studies should clarify the mechanisms through which GPR151 shapes motivated behavior and its relevance to neuropsychiatric conditions involving reward dysregulation.

### Non-CNS Roles of GPR151

The expression of GPR151 mRNA in peripheral tissues remains controversial. Early studies found low *GPR151* mRNA expression in peripheral organs, including the kidney and liver.^8^ More recent studies have found enriched expression in skeletal muscle and liver,^48^ which has inspired research into GPR151’s potential role in peripheral functions, including metabolic health and cancer. Notably, direct GPR151 protein expression in the periphery remains unverified, as studies primarily measure mRNA rather than functional protein. Given that mRNA levels only correlate with protein abundance, mRNA alone cannot be considered a reliable surrogate for protein expression.

#### GPR151 in Metabolic Health

A genome-wide meta-analysis linked GPR151 variants to BMI, obesity, and metabolic pathways.^69^ Predictive loss-of-function (pLOF) *GPR151* variants have been associated with multiple functional microbial pathways (e.g., homolactic fermentation, glucose and xylose degradation) and BMI-related conditions, including obesity, coronary artery disease, and type 2 diabetes.^70–72^ Specifically, the GPR151 pLOF variant (p.Arg95Ter) was associated with a 12% reduction in clinical obesity risk^70^ and a 14% decrease in risk of type 2 diabetes.^69–71^ These findings suggest that GPR151 loss-of-function may confer metabolic protection.

In addition to genetic screening data, studies have demonstrated state-dependent changes in GPR151 mRNA expression. In a diet-induced obesity model, GPR151 mRNA was upregulated in the hindbrain and pituitary gland but downregulated in the liver and subcutaneous white adipose tissue.^48^ An injection of insulin led to a significant downregulation of GPR151 mRNA in white adipose tissue, while an injection of glucagon led to an upregulation of transcript in the liver.^48^ Based on these observations, researchers hypothesized that GPR151 plays a role in adipocyte differentiation^71^ and hepatic gluconeogenesis,^48^ both of which are antiobesity therapeutic targets.^20,73,74^

To investigate GPR151’s function in adipocytes, Tanigawa and colleagues analyzed protein levels in the brain, subcutaneous adipose tissue and visceral adipose tissue. While detectable in adipose tissue, protein levels were substantially lower than the brain, raising questions about its functional relevance (see Supplemental Figure 27o in citation^71^). Further analysis using mouse 3T3-L1 and human Simpson-Golabi-Behmel Syndrome (SGBS) preadipocyte models showed that GPR151 expression remained low during adipocyte differentiation. However, siRNA-mediated deletion of GPR151 reduced adipogenesis markers, lipid accumulation, and lipolysis, suggesting that GPR151 deletion impairs adipocyte maturation.

Exploring GPR151’s role in hepatic glucogenesis, Bielczzyk-Maczynska and colleagues found that whole-body deletion of *GPR151* mRNA was associated with downregulation of genes related to glycolysis and gluconeogenesis in the liver.^48^ Pyruvate tolerance testing in mice with GPR151 deletion reported reduced blood glucose levels without a significant rise in blood lactate levels, indicating a liver-specific reduction in gluconeogenesis. Additionally, GPR151 deletion impaired basal and glucagon-mediated glucose production in primary hepatocytes. However, liver morphology and lipid metabolism remained unchanged, indicating a more selective role of GPR151 in glucose regulation.

Notably, these studies utilize a GPR151 knockout mouse line (GPR151tm1Dgen, RRID: MGI:3606630) that relies on Beta-galactosidase as a reporter. While initial studies demonstrated CNS-specific GPR151 expression,^75^ the emergence of subsequent studies showing broader mRNA expression in peripheral tissues raises concerns about the choice of Beta-galactosidase as a reporter, as it has known metabolic roles. While bacterial Beta-galactosidase differs from mammalian galactosidases and is generally considered an inert marker, it remains unclear whether the impact of Beta-galactosidase in reporter lines on metabolic function has been fully considered. Therefore, future studies should use alternative reporters to rule out any unintended metabolic effects. Moreover, given the roles of GPR151 described above in regulating inflammatory signaling, it also needs to be considered that GPR151 has an indirect role on metabolism through peripheral inflammatory processes.

To assess the therapeutic potential of GPR151, Gurtan and colleagues used CRISPR/Cas9 to delete *GPR151* and examined BMI-related changes.^76^ While GPR151 deletion resulted in no weight differences on standard chow, males gained more weight on a high-fat diet, indicating a possible sex-based effect of obesogenic diets.^76^ However, it is critical to note that the complete loss of function of GPR151 did not result in a clinically significant BMI change (>5%). Additionally, Gurtan et al. found that pLOF variants^69–71^ in human populations were unstable *in vitro* and showed no significant BMI reduction in heterozygous or homozygous carriers.^76^ Given these findings, GPR151 antagonism is unlikely to be a viable treatment for obesity or diabetes. GPR151 may still play a role in metabolism, but more research is needed to detail the extent.

#### GPR151 in Cancer

Another novel area of study for GPR151 has focused on its potential role in cancer. As GPR151 is shown to be acid-sensitive,^28^ a few studies investigated whether this protein is implicated in various cancers, as acidic environments can be generated by tumor metabolic changes and altered membrane-bound transporters. A few studies have indicated an upregulation of GPR151 proteins, as well as the presence of genetic variants, in patient samples with cancer.^77–79^

One potential explanation for the upregulation of GPR151 in cancer cells is the fact that cancer is often comorbid with chronic pain. In line with this assertion, genetic polymorphisms of mu opioid receptor 1 (OPRM1), which is highly colocalized with GPR151 expression in the habenula, are also associated with preoperative pain sensitivity in multiple types of cancer.^80^ Therefore, GPR151 and its variants in cancer studies may be more accurately attributed to cancer pain, rather than the development and maintenance of cancer. As most research related to GPR151 and cancer has only emerged over the past few years, this will be an important area of study to watch over the coming decade.

## Conclusion

Here we aggregate the prevailing literature surrounding the orphan receptor GPR151. Recent studies highlight GPR151’s importance in central and peripheral functions, with strong evidence demonstrating its roles in pain and inflammation, as well as reward and substance misuse. These functions appear to converge on common neural circuits, underscoring the receptor’s broad but specific impact on physiological and pathological processes.

Despite the growing interest in GPR151, much of the current knowledge of its anatomy, structure, and function remains preliminary. Key findings outlined in this review stem from a limited number of studies, with little to no independent replication. Furthermore, in the absence of an identified ligand, most insights into GPR151 have been derived from knockout models or genetically modified mouse strains. While these approaches are invaluable, they inherently disrupt native receptor function and may trigger compensatory biological changes, potentially obscuring GPR151’s true physiological role. This highlights the urgent need to identify an endogenous ligand and solve the receptor’s crystal structure, both of which are critical steps toward fully elucidating its mechanisms.

Despite significant gaps in our understanding, GPR151 represents a promising avenue for targeted therapeutic interventions. As a GPCR, it belongs to the largest and most pharmacologically tractable class of drug targets, making it a compelling candidate for drug development. What sets GPR151 apart is its highly restricted expression pattern, primarily localized to the habenula complex, spinal cord, and sensory ganglia—regions intricately linked to pain processing, addiction, and mood regulation. These conditions have long posed challenges for effective treatment, often due to the broad or nonspecific actions of existing therapies.

Given its proposed inhibitory function, modulating GPR151 could offer a novel strategy for dampening maladaptive neural activity, potentially mitigating chronic pain, substance dependence, and neuropsychiatric disorders. Moreover, its role in sensory pathways suggests broader implications for inflammatory and neuropathic pain conditions. Targeting GPR151 could thus pave the way for precision therapeutics with fewer off-target effects, addressing unmet medical needs in both neurological and inflammatory diseases. Unlocking its therapeutic potential will require deeper exploration of its endogenous ligand, signaling mechanisms, and downstream effects—but the opportunities for clinical impact are substantial.
